# Structure Properties, Acquisition Protocols, and Biological Activities of Oleuropein Aglycone

**DOI:** 10.3389/fchem.2018.00239

**Published:** 2018-08-13

**Authors:** Fangxue Xu, Yujuan Li, Mengmeng Zheng, Xiaozhi Xi, Xuelan Zhang, Chunchao Han

**Affiliations:** School of Pharmacy, Shandong University of Traditional Chinese Medicine, Jinan, China

**Keywords:** oleuropein aglycone, oleuropein, structure properties, acquisition protocols, biological activities

## Abstract

Oleuropein aglycone, which is the major phenolic component of extra virgin olive oil, is gaining popularity and importance in scientific and public communities. This paper summarizes the structure properties, acquisition protocols, and biological activities of oleuropein aglycone. There are three hydrolytic methods used to obtain oleuropein aglycone from oleuropein-enzymatic hydrolysis, acid hydrolysis, and acetal hydrolysis. Enzymatic hydrolysis can be achieved with exogenous enzymes and endogenous enzymes. In addition, the diverse pharmacological effects of oleuropein aglycone are summaried. These pharmacological effects include anti-Alzheimer's disease, anti-breast cancer, anti-inflammatory, anti-hyperglycemic, anti-oxidative, and lipid-lowering properties. Therefore, we can use hydrolysis and biological activities to study oleuropein aglycone in the future.

## Introduction

Oleuropein aglycone (OA), which is the chief phenolic substance of extra virgin olive oil (EVOO), is getting more and more global attention (Tangney et al., 2011; Vazquez-Martin et al., 2012; Lopez et al., 2014). It is derived from the deglycosylation of oleuropein that exists in the leaves and stone fruits of *Olea europaea* during the maturation period and obtained by squeezing (Rigacci et al., 2011; Rigacci and Stefani, 2015). In addition, OA, which is formed from oleuropein after the detachment of the glucose moiety, can be obtained by enzymatic hydrolysis, acid hydrolysis, and acetal hydrosis. Moreover, OA is a principal phenolic compound that is assimilated in the shape of oleuropein. After absorption, it is decomposed in the gastrointestinal tract of male human healthy volunteers. Urine was collected and detected (Visioli and Galli, 1998; Visioli et al., 2000b). OA is gaining increasing attention due to its biological properties such as anti-Alzheimer's disease, anti-breast cancer, anti-inflammatory, anti-hyperglycemic effect, anti-oxidative, and lipid-lowering properties. Research is necessary to get an overview of OA because of the increasing interest for its medicinal use. Therefore, our study reviewed the structural properties, extraction, and biological activities of OA for its significant role in the development of new drugs.

## Structural properties of OA

**Figure 1:**
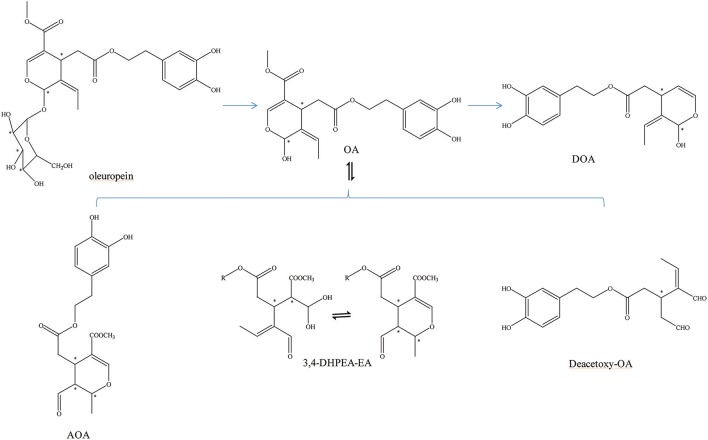
Other transformed structure forms of OA. ^*^represented the carbon atoms that are chiral.

OA can be transformed into many other structures (Figure 1). For example, OA and decarboxymethyl oleuropein aglycone (DOA) have identical dihydroxylated aromatic moieties in their structure. The difference between them is that a methoxycarbonyl group is presented on C-5 of the dihydropyrane ring of OA (Corominas-Faja et al., 2014). It can be found in several isomers due to the differentiated configuration of the iridoid moiety. However, the most abundant isomer in olive oil is always its monoaldehydic form. The three major diastereomers present were identified as (5S, 8R and 9S), (5S, 8S and 9S), and (5S, 8R and 9R). They are the aldehydic form of oleuropein aglycone (AOA) (Nikolaivits et al., 2017). In addition, there is an isomer of OA [3,4-(dihydroxyphenyl)ethanol elenolic acid ester,3,4-DHPEA-EA] and deacetoxy-OA (Smith et al., 2007; Pavia-Martins and Pinto, 2008). Additionally, Procopius et al. (2009) found four tautomer forms (glycine's a-d) of OA: the embolic forms, the dialdehydic forms, the ring-closed species, and a hydrated aldehydic derivative form.

## Extraction of OA

### Enzymatic hydrolysis

Enzymatic hydrolysis of oleuropein is an effective way to produce the resulting bioactive compound OA. The glycosidases are a widely scattered group of enzymes, which is of great biochemical, biomedical, and industrial importance. It functions by hydrolyzing the glycosidic bonds in oligos and polysaccharides (Visioli and Galli, 1998). There are two kinds of enzymes: exogenous enzymes and endogenous enzymes. The hydrolytic enzymes and detection methods of OA are summarized in Table 1.

**Table 1:** **List of proteins, whose abundance changes specifically upon infection with a *SIX* knockout as compared to wild-type Fol**.

**Enzymes**	**Source**	**Detection methods**	**References**
Recombinant β-glucosidase Mtbgl3a	*Myceliophthora thermophile*	LC-HRMS/MS and 1D & 2DNMR spectroscopy	Nikolaivits et al., 2017
Recombinant β-glycosidase (EcSβgly)	*Sulfolobus solfataricus*	TLC/GC/GC- MS/1HNMR	Briante et al., 2000
β-glycosidase	Almond	HPLC/LC-MS	Bouaziz and Sayadi, 2005
β-glycosidase	Almond	GC-MS	Rigacci et al., 2010; Grossi et al., 2013; Luccarini et al., 2015, 2016
β-glycosidase	Almond	TLC/MS	Walter et al., 1973
β-glycosidase	*Lactobacillus plantarumtype* strains	GC-MS	Ciafardini et al., 1994
β-glycosidase	*Aspergillus niger*	LC-MS/MS	Delgado-Povedano et al., 2017
β-glucosidase	Almond	1HNMR	Guiso and Marra, 2005
Endogenous enzyme	1-2 drops of the olive juice	1HNMR	Guiso and Marra, 2005
Endogenous β-glucosidase	Olive fruits	HPLC-MS-NMR	Brenes et al., 1999
β-glucosidase	Almond	HPLC/LC-MS	Jemai et al., 2008
β-glucosidase	Almond	HPLC-UV+(ESI)- MS/MS	Dell'Agli et al., 2008

**Figure 2:**
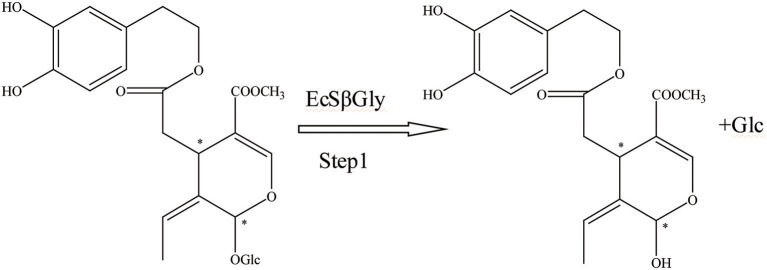
The hydrolysis progress of OA by recombinant enzyme (the EcSβgly enzyme from *Sulfolobus solfataricus*). ^*^represented the carbon atoms that are chiral.

**Figure 3:**
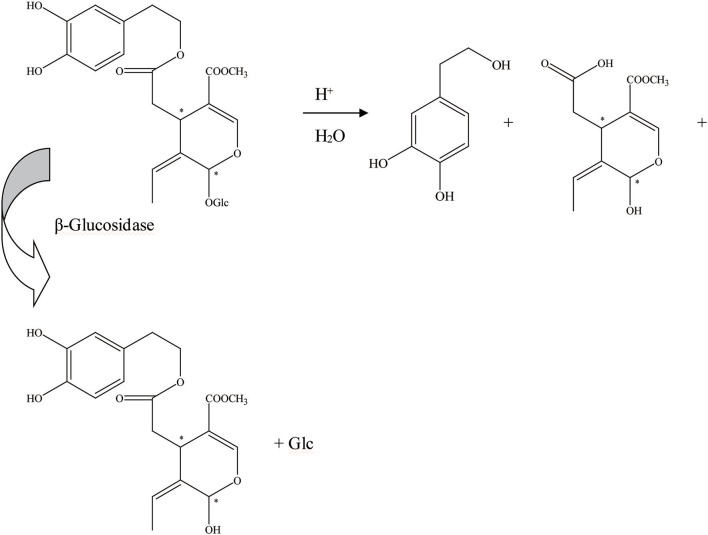
The hydrolysis progress of OA by β-glucosidase (the β-glucosidase from B21 *L.plantarum-*type strain). ^*^represented the carbon atoms that are chiral.

First, the β-glucosidase enzyme destroys the bond between glucose and the rest of the oleuropein molecule (Visioli et al., 2000b). The enzymatic hydrolysis of oleuropein by β-glucosidase activity was already considered to obtain glucose and OA (Limiroli et al., 1995, 1996). Utilization of a recombinant β-glucosidase Mtbgl3a from *Myceliophthora thermophila* and three major diastereomers present in the β-glucosidase-catalyzed reaction mixture were identified as (5S, 8R, and 9S), (5S, 8S,and 9S), and (5S, 8R, and 9R). The identification of the hydrolysis products was performed by liquid chromatogram-high resolution mass spectrometer/mass spectrometer (LC-HRMS/MS) and 1D & 2D nuclear magnetic resonance (NMR) experiments (Nikolaivits et al., 2017). The multiple β-glycosidase (EcSβgly) from *Sulfolobus solfataricus* was utilized to complete three enzymatic hydrolysis of commercial oleuropein at two temperatures (Figure 2). The structure was determined by thin-layer chromatography/gas chromatography/gas chromatography-mass spectrometer/1H NMR (TLC/GC/GC-MS/1HNMR) (Briante et al., 2000). Bouaziz and Sayadi (2005) conducted enzymatic hydrolysis using β-glycosidase from almonds. The biotransformation of the olive leaf extract by β-glucosidase showed a high concentration of OA after 2 h of incubation time. Moreover, OA is obtained by enzymatic hydrolysis, which is performed by β-glucosidase-induced deglucosylation of the iridoid glycoside moiety of oleuropein (Konno et al., 1999). Rigacci et al. (2010), Luccarini et al. (2015, 2016), and Grossi et al. (2013) performed hydrolysis according to Konno et al. (1999) with little modification. GC-MS analysis showed that glycated oleuropein was not found in the precipitate. OA was obtained by enzymatic hydrolysis of oleuropein and by utilizing β-glucosidase. Our preparation of aglycone was subjected to several purification steps and was chromatographically pure (TLC) (Walter et al., 1973). Oleuropein was discovered to be catalytically hydrolyzed by the β-glucosidase (EC 3.2.1.2.1) derived from oleuropeinolytic *Lactobacillus plantarum*-type strains. The β-glucosidases produced by the B17, B20, and B21 *L. plantarum*-type strains were equally active on oleuropein. Research demonstrated the ability of the B21 *L. plantarum*-type strain to hydrolyze oleuropein via β-glucosidase production to aglycone and other simpler compounds, and among which β-3,4-dihydroxyphenylethanol was identified (Figure 3) (Ciafardini et al., 1994). In addition, other enzymes can be used in the hydrolysis of oleuropein to obtain OA and other subsequent products. Three of the most used hydrolases (hemicellulase and β-glucosidase from *A. niger* and β-glucosidase from almonds) were assayed to catalyze the hydrolytic process. In the end, β-glucosidase from *A. niger* was picked among the three enzymes used for the following studies because of its shorter reaction time. Furthermore, the dynamics of the ultrasound-assisted enzymatic hydrolysis (USAEH) was detected by the analysis of the target materials by means of LC-MS/MS. After optimizing the USAEH method, a dynamics study indicated that the hydrolysis response of oleuropein was accomplished in merely 18.75 min (Delgado-Povedano et al., 2017).

**Figure 4:**
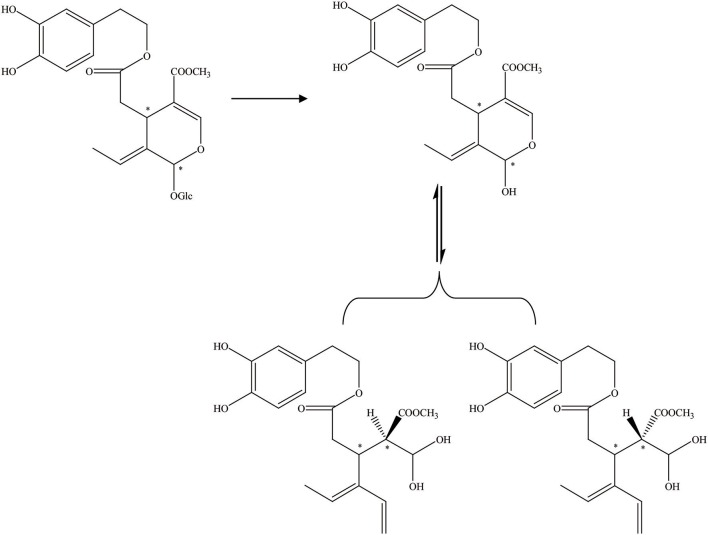
The hydrolysis progress of OA by endogenous β-glucosidase (the β-glucosidase from olive juice). ^*^represented the carbon atoms that are chiral.

Furthermore, aglycons that arise from glucosides can also be achieved by endogenous β-glucosidase hydrolysis (Brenes et al., 1999). In addition, the oleuropein hydrolytic conversion catalyzed by an endogenous enzyme of the olive fruit has been evaluated by Bianco et al. (1999). The endogenous β-glycosidase comes from olive juice. There are two schemes for the hydrolysis of oleuropein. Thirty milligrams of oleuropein was dissolved in 0.7 mL of D2O and 1–2 drops of olive juice was added to the solution (Figure 4). One hundred milligrams of oleuropein was dissolved in 20 mL of D2O/CDCl3 (1:1) mixture, and 2 drops of olive juice were added. Oleuropein hydrolysis progress could be executed by an endogenous β-glycosidase derived from the olive ripening period or by squeezing. During this progress, the aglycon section of the molecule could be obtained (Soler-Rivas et al., 2000).

**Figure 5:**
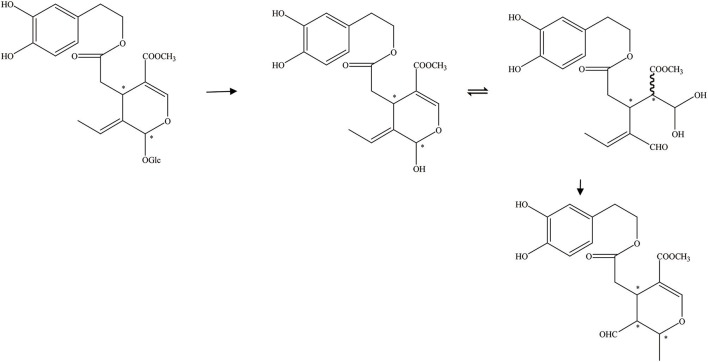
The hydrolysis of OA by commercial enzyme (the commercial enzyme from almonds). ^*^represented the carbon atoms that are chiral.

**Figure 6:**
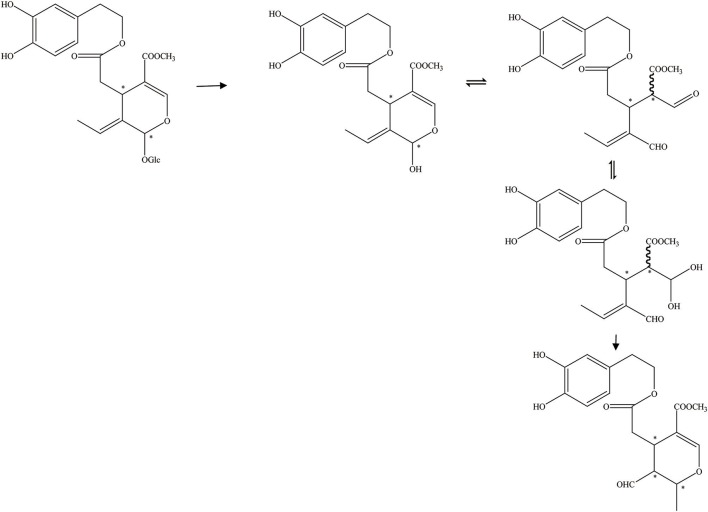
The hydrolysis progress of OA by endogenous enzyme (the endogenous enzyme from olive juice). ^*^represented the carbon atoms that are chiral.

In addition, Guiso and Marra (2005) studied whether the hydrolysis products acquired from a commercial enzyme and those received from an endogenous enzyme are equal. There are two methods of enzymatic hydrolysis to hydrolyze oleuropein. The commercial enzyme utilized in the hydrolytic process has been summarized in Figure 5. The commercial enzyme was separated from almonds. In contrast, the hydrolytic scheme by endogenous enzymes has been summed up in Figure 6. The endogenous enzyme was acquired using 1–2 drops of the same olive juice. They found that the OA acquired from the enzymatic hydrolysis is actually a mixture of substances with different structures. The OA was studied by traditional methodologies (TLC) and detected by 1HNMR experiment (Guiso and Marra, 2005).

### Acid hydrolysis

In addition, acid hydrolysis is another important pattern to obtain OA. OA is extracted from the olive leaf by acid hydrolysis. One gram of the ethyl acetate extract was dissolved in 10 mL of MeOH/H_2_O (4:1) mixture in a sealed vial. The solution was hydrolyzed at 100°C for 1 h using 5 mL of a 2 M HCl solution (Jemai et al., 2008). Olives were placed in vessels and combined with 12 L of brine (8% NaCl and 0.4% acetic acid) for spontaneous anaerobic and aerobic fermentations. The acid hydrolysis of the initial glucosides and aglycons was the main reaction that occurred during the fermentation process (Pérez-Trujillo et al., 2010).

### Acetal hydrolysis

**Figure 7:**
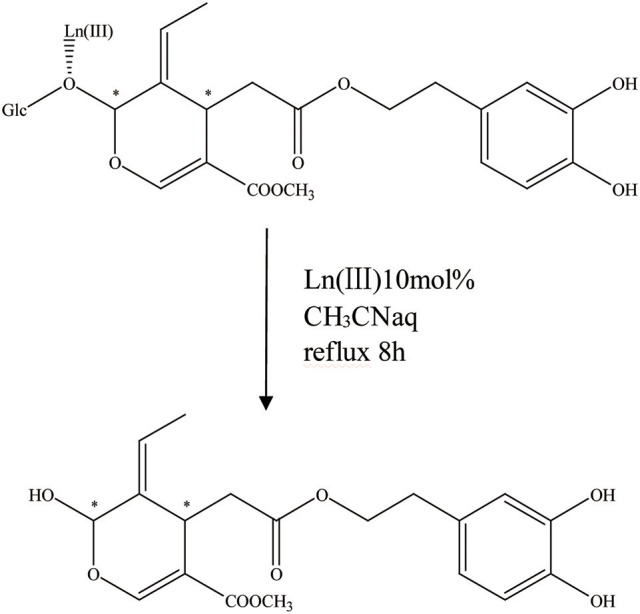
The acetal hydrolysis progress of OA. ^*^represented the carbon atoms that are chiral.

It is apparent that the aglycone form can also be acquired by acetal hydrolysis, which is similar to the natural glucosidase enzyme reaction (Figure 7). Oleuropein was dissolved in aqueous CH_3_CN carried with Er(OTf)3 and refluxed for 8 h at 80°C. In the end, the hydrolysate was cooled, 5 mL of water was added, and the mixture was extracted with CH_2_Cl_2_. After drying on Na_2_SO_4_, the organic solvent was removed in a vacuum and the crude product was purified by flash chromatography, and the aglycon as a mixture of three isomers was obtained. They were all characterized by high-performance liquid chromatography (HPLC), LC/MS/electrospray ionization (ESI), and 1HNMR (total yield = 70%) (Procopius).

### Detection methods

In addition to the above detection methods, there are also many test methods. For example, Chen et al. (2011) quantified the identified phenolics using HPLC-UV. A reverse-phase HPLC technique was utilized to evaluate and quantify OA that existed in the hydrolysate extract (Jemai et al., 2008). OA was tested qualitatively and quantitatively by HPLC-UV and ESI-MS/MS (Dell'Agli et al., 2008). The structure of the newly isolated material was monitored using proton and carbon magnetic resonance tests (Pavia-Martins and Pinto, 2008). For quantitative analysis of OA, ESI-MS/MS was used (Dell'Agli et al., 2006). Pérez-Bonilla et al. (2014) detected OA by HPLC-DAD-DPPH/ABTS technique. Pérez-Trujillo et al. (2010) devoted themselves to exploring the unknown isomeric forms of OA by HPLC-DAD-SPE-NMR/MS techniques. The extract was evaluated quantitatively by high-performance liquid chromatography along with electrospray ionization-quadrupole time-of-flight mass spectrometry (HPLC–ESI-QTOF-MS) (Quirantes-Piné et al., 2013). The metabolized mixture from the EVOO-crude phenolic extracts (PE) in the medium and cytoplasm of both SW480 and HT29 human colon adenocarcinoma cell lines was monitored by nano-liquid chromatography (nanoLC) along with ESI-TOF-MS. The chief compounds determined from EVOO-PE were hydroxylated luteolin and decarboxymethyl OA (Fernández-Arroyo et al., 2012). HPLC-MS-NMR was used to analyze the OA (Brenes et al., 1999). Research found that OA was the main compound in extra virgin olive oils by HPLC and capillary zone electrophoresis (CZE) (Bonoli et al., 2004). Qualitative analysis was conducted in the atmospheric pressure chemical ionization (APCI)-MS scan mode (m/z 100–600), whereas quantitative detection was performed by APCI-MS/MS (Bisignano et al., 2014).

## Biological activity of OA

### Anti-alzheimer's disease effect

Alzheimer's disease (AD) is a high social impact disease that represents approximately 55–60% of all dementias and affects 6% of aged people. AD is marked by cognitive degradation with a progressive damage of activities of daily living (Valls-Pedret et al., 2012).

Amyloid β (Aβ) deposits and oligomers are also found in AD. Regarding Aβ-induced neurodegeneration, recently reported findings indicate that OA administration to TgCRND8 (Tg) mice can improve memory and behavioral performance. The mechanisms may be interfering with Aβ aggregation and restoring the transgene-associated different dysfunctional aspects in the brain of our mouse model of AD (Casamenti et al., 2015). In addition, OA provides neuroprotection to cultured neuronal cells and murine models by intervening with Aβ aggregation, decreasing aggregate cytotoxicity, and counteracting related neuroinflammation (Luccarini et al., 2014). In addition, Grossi et al. (2013) found that the administration of OA could improve memory and behavioral performance of AD mice by interfering with Aβ aggregation and signaling epigenetic pathways. In addition, they showed that OA was positive against glutaminylcyclase-catalyzed pE3-Aβ generation reducing enzyme expression and interfered with both Aβ42 and pE3-Aβ aggregations after 1 year. They suggested that OA administration could be regarded as a new way to prevent and cure AD (Luccarini et al., 2015). The level of OA interfering with Aβ proteotoxicity *in vivo* was also evaluated. In the end, their results showed that a reasonable supply of OA can be useful against AD (Diomede et al., 2013). It can be concluded that OA offsets amyloid aggregation and the toxicity affects different pathways: amyloid precursor protein processing, Aβ peptide and tau aggregation, autophagy impairment, and neuroinflammation (Martorell et al., 2016).

In addition, autophagic response is considered as an important action against AD. Rigacci et al. (2015) investigated the mechanisms of autophagy induction by OA-utilizing cultured neuroblastoma cells and an OA-fed mouse model of amyloid Aβ deposition. They showed that OA induced autophagy in cultured cells via the Ca2+-CAMKKβ-AMPK axis. OA administration improved memory dysfunction, raised a crucial autophagic response in the cortex, and facilitated the proliferation of newborn cells in the subgranular zone of the dentategyrus of the hippocampus (Grossi et al., 2014). OA supplementation was found to obviously increase the cognitive ability of Tg mice. OA significantly decreased the Aβ42 and pE-3 Aβ plaque area and the number in the cortex region. Homologous autophagy induction was obtained in the brain cortex of differently disposed mice (Pantano et al., 2017).

Moreover, speaking of AD, Tau protein aggregation into fibrillary tangles leads to intraneuronal and glial lesions. OA was found to be more active than the reference tau aggregation inhibitor methylene blue on both wild-type and P301L tau proteins. This played a role in inhibiting fibrillization at low concentrations. It might offer a new direction for the treatment of neurodegenerative tauopathies (Daccache et al., 2011).

### Anti-breast cancer effect

Research shows that anti-cancer function can occur by inhibiting the expression of the lipogenic enzyme FASN in Human Epidermal Growth Factor Receptor2 (HER2)-overexpressing breast carcinoma cells, and thus offers a previously unrecognized mechanism for EVOO-related cancer preventive effects (Menendez et al., 2008a). In addition, OA synergizes with trast usumab-sensitive breast cancer cell lines. OA was the most promising EVOO phenolic in reducing breast cancer cell activity. OA immediately down-regulates both the HER2 expression and the tyrosine kinase activity-cultured HER2-overexpressing breast cancer cells. Therefore, it was concluded that OA could directly regulate HER2-driven breast cancer disease (Menendez et al., 2007). In addition, it was demonstrated that OA inhibited fatty acid synthase and increased the accumulation of malonyl-CoA that has been shown to indirectly inhibit transcription of HER1 (Menendez et al., 2008b).

### Anti-inflammatory effect

Inflammation is a complex immune response to pathogens, damaged cells, or irritants, and enables the survival during infection or injury and maintains tissue homeostasis under a variety of noxious conditions (Medzhitov, 2010). OA could show an effect against secondary events of reperfusion injury. The treatment with OA largely decreased the apoptotic cell death after splanchnic arterial occlusion (SAO) shock, which suggests that protection from apoptosis may be a necessity for anti-inflammatory approaches (Campolo et al., 2013). In addition, OA plays an anti-inflammatory role during chronic inflammation and improves tissue damage associated with collagen-induced arthritis (Impellizzeri et al., 2011b). Miles and Zoubouli (2005) found that OA inhibited interleukin-1 production by 80% in the culture supernatants. Furthermore, OA associated with a mouse model of spinal cord injury (SCI) and carrageenan-induced pleurisy was studied. Overall, the OA treatment significantly decreased the SCI-induced spinal cord tissue alterations and promoted motor function. The results imply that the anti-inflammatory effect of OA may be helpful in the therapy of SCI, trauma, and inflammation (Impellizzeri et al., 2011a, 2012). In addition, Procopius et al. (2009) discovered that the anti-inflammatory activity of OA could be achieved by inhibiting cyclooxygenase (COX) enzymes. Therefore, OA can play an effective role in anti-inflammatory activities.

### Anti-hyperglycemic effect

Pancreatic amyloid deposits of amylin are characteristic indications of type II diabetes. The effect on amylin aggregation and the cytotoxicity of OA were investigated. OA had the ability to intervene with the early steps of human islet amyloid polypeptide (hIAPP) aggregation and hinder the appropriate reorganization of the polypeptide chain and the appearance of the most cytotoxic species. In addition, OA could delay the proliferation of harmless amyloid fibrils that are structurally different from those grown in the absence of polyphenol. The authors concluded that OA could prevent or retard the development of type II diabetes (Rigacci et al., 2010). In addition, autophagy dysfunction plays an important role in type II diabetes. Rigacci et al. (2015) showed that OA triggered autophagy in cells via the Ca^2+^-CAMKKβ-AMPK axis. The results suggest the existence of a common molecular mechanism underlying the health effects of these materials against type II diabetes.

### Anti-oxidative effect

The administration of OA-rich oils was dose-dependently related to a decrease in urinary excretion of 8-iso-PGF2a, which is a biomarker of oxidative stress. The authors concluded that OA may have an anti-oxidative effect (Visioli et al., 2000a). In addition, Pérez-Bonilla et al. (2014) found that OA could display a higher anti-oxidative effect against the free radical DPPH than the reference 2,6-di-tert-butyl-hydroxytoluene (BHT). Oxidation of low-density lipoproteins (LDL) is deemed to increase the incidence of atherogenesis, which is a potential cause of coronary heart disease. OA was reported to protect LDL in plasma against oxidation (Leenen et al., 2002). In summary, OA can inhibit copper sulfate-induced oxidation, and when taken together with other dietary anti-oxidants, it can prevent lipoprotein oxidation, which is considered relevant in the development of atherosclerotic disease (Visioli et al., 1995).

### Lipid-lowering effect

The hypocholesterolemic effect of OA-rich extracts might lie within their capacity to reduce serum TC, TG, and LDL-C levels and retard the lipid peroxidation process and enhance anti-oxidant enzyme activity (Jemai et al., 2008). In addition, research indicates that OA plays a protective role against cardiovascular risk via the reduction of adhesion molecules involved in early atherogenesis (Dell'Agli et al., 2006). Furthermore, they show that OA is the agonist of both the transient receptor potential ankyrin subtype 1 (TRPA1) and the transient receptor potential vanilloid subtype 1 (TRPV1). OA enhances uncoupling protein 1 expression in interscapular brown adipose tissue coupled with a decrease in the visceral fat mass of HF diet-induced obese rats. The mechanism of its function has increased the noradrenaline secretion via β-adrenergic action following TRPA1 and TRPV1 activations (Oi-Kano et al., 2017).

## Conclusion

OA, which is an important phenolic component, is formed from oleuropein after the detachment of the glucose moiety. In this paper, we summarize three hydrolytic methods to obtain OA from oleuropein including enzymatic hydrolysis, acid hydrolysis, and acetal hydrolysis. Enzymatic hydrolysis can be achieved by exogenous enzymes and endogenous enzymes. Moreover, we also summarize the biological activities of OA. Its pharmacological actions include anti-Alzheimer's disease, anti-breast cancer, anti-inflammatory, anti-hyperglycemic, anti-oxidative, and lipid-lowering properties. This review will act as a reference for people to research and develop new drugs. To date, only a little is known about the wonders of this component. It still has many secrets for us to discover and more research is needed on this active ingredient.

## Author contributions

FX prepared the literature. YL and MZ revised the literature and prepared tables. XX revised the manuscript and prepared figures. XZ put forward valuable opinions on the ideas and structure of the paper based on the original paper. XZ provided valuable advice on the revision of the paper. All authors read and approved the final manuscript.

### Conflict of interest statement

The authors declare that the research was conducted in the absence of any commercial or financial relationships that could be construed as a potential conflict of interest.

## References

[B1] Bianco A. D. Piperno A. Romeo G. Uccella N. (1999). NMR experiments of oleuropein biomimetic hydrolysis. J. Agric. Food Chem. 47, 3665–3668. 10.1021/jf981241h 10552701

[B2] Bisignano C. Filocamo A. Ginestra G. Giofre S. V. Navarra M. Romeo R. (2014). 3,4-DHPEA-EA from *Olea europaea* L. is effective against standard and clinical isolates of *Staphylococcus* sp*. Ann. Clin. Microbiol. Antimicrob* 13:24 10.1186/1476-0711-13-24 PMC4107751 24986240

[B3] Bonoli M. Bendini A. Cerretani L. Lercker G. Toschi T. G. (2004). Qualitative and semiquantitative analysis of phenolic compounds in extra virgin olive oils as a function of the ripening degree of olive fruits by different analytical techniques. J. Agric. Food Chem. 52, 7026–7032. 10.1021/jf048868m 15537313

[B4] Bouaziz M Sayadi S. (2005). Isolation and evaluation of antioxidants fromleaves of a Tunisian cultivar olive tree. Eur. J. Lipid Sci. Technol. 107, 497–504. 10.1002/ejlt.200501166

[B5] Brenes M. García A. García P. Rios J. J. (1999). Garrido A. Phenolic compounds in Spanish olive oils. J Agric Food Chem. 47, 3535–3540. 10.1021/jf990009o 10552681

[B6] Briante R. La Cara F. Febbraio F. Barone R. Piccialli G. Carolla R.. (2000). Hydrolysis of oleuropein by recombinant β-glycosidase from hyperthermophilic archaeon *Sulfolobus solfataricus* immobilised on chitosan matrix. J. Biotechnol. 77, 275–286. 10.1016/S0168-1656(99)00219-9 10682286

[B7] Campolo M. Di Paola R. Impellizzeri D. Crupi R. Morittu V. M. Procopius A.. (2013). Effects of a polyphenol present in olive oil, oleuropein aglycone, in a murine model of intestinal ischemia/reperfusion injury. J. Leukoc. Biol. 93, 277–287. 10.1189/jlb.0712317 23233730

[B8] Casamenti F. Grossi C. Rigacci S. Pantano D. Luccarini I. Stefani M. (2015). oleuropein aglycone: a possible drug against degenerative conditions *in vivo* evidence of its effectiveness against Alzheimer's disease. J. Alzheimers Dis. 45, 679–688. 10.3233/JAD-142850 25649656

[B9] Chen Y. Whitehill J. G. Bonello P. (2011). Differential response in foliar chemistry of three ash species to emerald ash borer adult feeding. J. Chem. Ecol. 37, 29–39. 10.1007/s10886-010-9892-1 21153046

[B10] Ciafardini G. Marsilio V. Lanza B. Pozzi N. (1994). Hydrolysis of oleuropein by *Lactobacillus plantarum* strains associated with olive fermentation. Appl. Environ. Microbiol. 60, 4142–4147. 16349442 10.1128/aem.60.11.4142-4147.1994 PMC201948

[B11] Corominas-Faja B. Santangelo E. Cuyàs E. Micol V. Joven J. Ariza X.. (2014). Computer-aided discovery of biological activity spectra for anti-aging and anti-cancer olive oil oleuropeins. Aging 6, 731–741. 10.18632/aging.100691 25324469 PMC4221918

[B12] Daccache A. Lion C. Sibille N. Gerard M. Slomianny C. Lippens G.. (2011). Oleuropein and derivatives from olives as Tau aggregation inhibitors. Neurochem. Int. 58, 700–707. 10.1016/j.neuint.2011.02.010 21333710

[B13] Delgado-Povedano M. D. Priego-Capote F. Luque de Castro M. D. (2017). Selective ultrasound-enhanced enzymatic hydrolysis of oleuropein to its aglycon in olive (Oleaeuropaea L) leaf extracts. Food Chem. 220, 282–288. 10.1016/j.foodchem.2016.10.011 27855900

[B14] Dell'Agli M. Fagnani R. Mitro N. Scurati S. Masciadri M. Mussoni L.. (2006). Minor components of olive oil modulate proatherogenic adhesion molecules involved in endothelial activation. J. Agric. Food Chem. 54, 3259–3264. 10.1021/jf0529161 16637682

[B15] Dell'Agli M. Maschi O. Galli G. V. Fagnani R. Dal Cero E. Caruso D.. (2008). Inhibition of platelet aggregation by olive oil phenols via cAMP-phosphodiesterase. Br. J. Nutr. 99, 945–951. 10.1017/S0007114507837470 17927845

[B16] Diomede L. Rigacci S. Romeo M. Stefani M. Salmona M. (2013). Oleuropein aglycone protects transgenic C elegans strains expressing Aβ42 by reducing plaque load and motor deficit. PLoS ONE 8:e58893. 10.1371/journal.pone.0058893 23520540 PMC3592812

[B17] Fernández-Arroyo S. Gómez-Martínez A. Rocamora-Reverte L. Quirantes-Piné R. Segura-Carretero A. Fernández-Gutiérrez A.. (2012). Application of nanoLC-ESI-TOF-MS for the metabolomic analysis of phenolic compounds from extra-virgin olive oil in treated colon-cancer cells. J. Pharm. Biomed. Anal. 63, 128–134. 10.1016/j.jpba.2012.01.033 22365054

[B18] Grossi C. Ed Dami T. Rigacci S. Stefani M. Luccarini I. Casamenti F. (2014). Employing Alzheimer disease animal models for translational research: focus on dietary components. Neurodegener. Dis. 13, 131–134. 10.1159/000355461 24192327

[B19] Grossi C. Rigacci S. Ambrosini S. Ed Dami T. Luccarini I. Traini C. (2013). The polyphenol oleuropein aglycone protects TgCRND8 mice against Aβ plaque pathology. PLoS ONE 8:e71702 10.1371/journal.pone.0071702 23951225 PMC3738517

[B20] Guiso M. Marra C. (2005). Highlights in oleuropein aglycone structure. Nat. Prod. Res. 19, 105–109. 10.1080/14786410410001696147 15715252

[B21] Impellizzeri D. Esposito E. Mazzon E. Paterniti I. Di Paola R. Bramanti P.. (2012). The effects of a polyphenol present in olive oil, oleuropeinaglycone, in an experimental model of spinal cord injury in mice. Biochem. Pharmacol. 83, 1413–1426. 10.1016/j.bcp.2012.02.001 22342994

[B22] Impellizzeri D. Esposito E. Mazzon E. Paterniti I. Paola R. D. Bramanti P.. (2011a). The effects of oleuropein aglycone, an olive oil compound, in a mouse model of carrageenan-induced pleurisy. Clin. Nutr. 30, 533–540. 10.1016/j.clnu.2011.02.004 21411195

[B23] Impellizzeri D. Esposito E. Mazzon E. Paterniti I. Paola R. D. Morittu V. M.. (2011b). Oleuropein aglycone, an olive oil compound, ameliorates development of arthritis caused by injection of collagen type II in mice. J. Pharmacol. Exp. Ther. 339, 859–869. 10.1124/jpet.111.182808 21880869

[B24] Jemai H. Bouaziz M. Fki I. El Feki A. Sayadi S. (2008). Hypolipidimic and antioxidant activities of oleuropein and its hydrolysis derivative-rich extracts from Chemlali olive leaves. Chem. Biol. Interact. 176, 88–98. 10.1016/j.cbi.2008.08.014 18823963

[B25] Konno K. Hirayama C. Yasui H. Nakamura M. (1999). Enzymatic activation of oleuropein: a protein crosslinker used as a chemical defense in the privet tree. Proc. Natl. Acad. Sci. U.S.A. 96, 9159–9164. 10.1073/pnas.96.16.9159 10430912 PMC17749

[B26] Leenen R. Roodenburg A. J. Vissers M. N. Schuurbiers J. A. van Putte K. P. Wiseman S. A.. (2002). Supplementation of plasma with olive oil phenols and extracts: influence on LDL oxidation. J. Agric. Food Chem. 50, 1290–1297. 10.1021/jf010968u 11853520

[B27] Limiroli R. Consonni R. Ottolina G. Marsilio V. Bianchi G. Zetta L. (1995). 1H and 13C NMR characterisation ofnew oleuropeinaglycones. J. Chem. Soc. Perkin Trans. 1, 1519–1523. 10.1002/chin.199541242

[B28] Limiroli R. Consonni R. Ottolina G. Marsilo V. Bianchi G. Zetta L. (1996). 1H NMR study of phenolics in vegetation water ofthree cultivars of Oleaeuropaea: similarities and differences. J. Chem. Soc. Perkin. Trans. 44, 2040–2048. 10.1021/jf9507349

[B29] Lopez S. Bermudez B. Montserrat-de la Paz S. Jaramillo S. Varela L. M. Ortega-Gomez A.. (2014). Membrane composition and dynamics: a target of bioactive virgin olive oil constituents. Biochim. Biophys. Acta 1838, 1638–1656. 10.1016/j.bbamem.2014.01.007 24440426

[B30] Luccarini I. Ed Dami T. Grossi C. Rigacci S. Stefani M. Casamenti F. (2014). Oleuropein aglycone counteracts Aβ42 toxicity in the rat brain. Neurosci. Lett. 558, 67–72. 10.1016/j.neulet.2013.10.062 24211687

[B31] Luccarini I. Grossi C. Rigacci S. Coppi E. Pugliese A. M. Pantano D.. (2015). Oleuropein aglycone protects against pyroglutamylated-3 amyloid-ß toxicity: biochemical, epigenetic and functional correlates. Neurobiol. Aging 36, 648–663. 10.1016/j.neurobiolaging.2014.08.029 25293421

[B32] Luccarini I. Pantano D. Nardiello P. Cavone L. Lapucci A. Miceli C.. (2016). The polyphenol oleuropein aglycone modulates the PARP1-SIRT1 Interplay: an *in vitro* and *in vivo* study. J. Alzheimers Dis. 54, 737–750. 10.3233/JAD-160471 27567859

[B33] Martorell M. Forman K. Castro N. Cap,ó X. Tejada S. Sureda A. (2016). Potential therapeutic effects of oleuropein aglycone in Alzheimer's disease. Curr. Pharm. Biotechnol. 17, 994–1001. 10.2174/1389201017666160725120656 27455905

[B34] Medzhitov R. (2010). Inflammation 2010: new adventures of an old flame. Cell 140, 771–776. 10.1016/j.cell.2010.03.006 20303867

[B35] Menendez J. A. Vazquez-Martin A. Colomer R. Brunet J. Carrasco-Pancorbo A. Garcia-Villalba R.. (2007). Olive oil's bitter principle reverses acquired autoresistance to trastuzumab (Herceptin) in HER2-overexpressing breast cancer cells. BMC Cancer 7:80. 10.1186/1471-2407-7-80 17490486 PMC1878493

[B36] Menendez J. A. Vazquez-Martin A. Garcia-Villalba R. Carrasco-Pancorbo A. Oliveras-Ferraros C. Fernandez-Gutierrez A.. (2008a). tabAnti-HER2 (erbB-2) oncogene effects of phenolic compounds directly isolated from commercial Extra-Virgin Olive Oil (EVOO). BMC Cancer 8:377. 10.1186/1471-2407-8-377 19094209 PMC2626601

[B37] Menendez J. A. Vazquez-Martin A. Oliveras-Ferraros C. Garcia-Villalba R. Carrasco-Pancorbo A. Fernandez-Gutierrez A.. (2008b). Analyzing effects of extra-virgin olive oil polyphenols on breast cancer-associated fatty acid synthase protein expression using reverse-phase protein microarrays. Int. J. Mol. Med. 22, 433–439. 10.1016/S0304-3800(03)00229-1 18813848

[B38] Miles E. A. Zoubouli P. (2005). Differential anti-inflammatory effects of phenolic compounds from extra virgin olive oil identified in human whole blood cultures. Nutrition 21, 389–394. 10.1016/j.nut.2004.06.031 15797683

[B39] Nikolaivits E. Termentzi A. Skaltsounis A. L. Fokialakis N. Topakas E. (2017). Enzymatic tailoring of oleuropein from Oleaeuropaea leaves and product identification by HRMS/MS spectrometry. J. Biotechnol. 253, 48–54. 10.1016/j.jbiotec.2017.05.020 28576392

[B40] Oi-Kano Y. Iwasaki Y. Nakamura T. Watanabe T. Goto T. Kawada T.. (2017). Oleuropein aglycone enhances UCP1 expression in brown adipose tissue in high-fat-diet-induced obese rats by activating β-adrenergic signaling. J. Nutr. Biochem. 40, 209–218. 10.1016/j.jnutbio.2016.11.009 27951473

[B41] Pavia-Martins F. Pinto M. (2008). Isolation and characterization of a new hydroxytyrosol derivative from olive (Oleaeuropaea) leaves. J. Agric. Food Chem. 56, 5582–5588. 10.1021/jf800698y 18582082

[B42] Pantano D. Luccarini I. Nardiello P. Servili M. Stefani M. Casamenti F. (2017). Oleuropein aglycone and polyphenols from olive mill waste water ameliorate cognitive deficits and neuropathology. Br. J. Clin. Pharmacol. 83, 54–62. 10.1111/bcp.12993 27131215 PMC5338135

[B43] Pérez-Bonilla M. Salido S. van Beek T. A. Altarejos J. (2014). Radical-scavenging compounds from olive tree (Oleaeuropaea L) wood. J. Agric. Food Chem. 62, 144–151. 10.1021/jf403998t 24328093

[B44] Pérez-Trujillo M. Gómez-Caravaca A. M. Segura-Carretero A. Fernández-Gutiérrez A. Parella T. (2010). Separation and identification of phenolic compounds of extra virginolive oil from *Oleaeuropaea* L. by HPLC-DAD-SPE-NMR/MS. Identification of a newdiastereoisomer of the aldehydic form of oleuropeinaglycone. J. Agric. Food Chem. 58, 9129–9136. 10.1021/jf101847e 23654238

[B45] Procopius A. Alcaro S. Nardi M. Oliverio M. Ortuso F. Sacchetta P. (2009). Synthesis, biological evaluation, and molecular modeling of oleuropein and its semisynthetic derivatives as cyclooxygenase inhibitors. J. Agric. Food Chem. 57, 11161–11167. 10.1021/jf9033305 19908866

[B46] Quirantes-Piné R. Zurek G. Barrajón-Catalán E. Bäßmann C. Micol V. Segura-Carretero A.. (2013). A metabolite-profiling approach to assess the uptake and metabolism of phenolic compounds from olive leaves in SKBR3 cells by HPLC-ESI-QTOF-MS. J. Pharm. Biomed. Anal. 72, 121–126. 10.1016/j.jpba.2012.09.029 23146235

[B47] Rigacci S. Stefani M. (2015). Nutraceuticals and amyloid neurodegenerative diseases: a focus on natural phenols. Expert Rev. Neurother. 15, 41–52. 10.1586/14737175.2015.986101 25418871

[B48] Rigacci S. Guidotti V. Bucciantini M. Daniela N. Relini A. Berti A.. (2011). Aβ(1-42) aggregates into non-toxic amyloid assemblies in the presence of the natural polyphenol oleuropeinaglycon. Curr. Alzheimer Res. 8, 841–852. 10.2174/156720511798192682 21592051

[B49] Rigacci S. Guidotti V. Bucciantini M. Parri M. Nediani C. Cerbai E.. (2010). Oleuropein aglycon prevents cytotoxic amyloid aggregation of human amylin. J. Nutr. Biochem. 21, 726–735. 10.1016/j.jnutbio.2009.04.010 19616928

[B50] Rigacci S. Miceli C. Nediani C. Berti A. Cascella R. Pantano D.. (2015). Oleuropein aglycone induces autophagy via the AMPK/mTORsignalling pathway: a mechanistic insight. Oncotarget 6, 35344–35357. 10.18632/oncotarget.6119 26474288 PMC4742109

[B51] Smith A. B. Sperry J. B. Han Q. (2007). Syntheses of (-)-oleocanthal, a natural NSAID found in extra virgin olive oil, the (-)-deacetoxy-oleuropeinaglycone, and related analogues. J. Org. Chem. 72, 6891–6900. 10.1021/jo071146k 17685574

[B52] Soler-Rivas C. Espin J. C. Wichers H. J. (2000). Oleuropein and related compounds. J. Sci. Food Agric. 80, 1013–1023. 10.1002/(SICI)1097-0010(20000515)80:7<1013::AID-JSFA571>3.0.CO;2-C

[B53] Tangney C. C. Kwasny M. J. Li H Wilson R. S. Evans D. A. Morris M. C. (2011). Adherence to a Mediterranean-type dietary pattern and cognitive decline in acommunitypopulation. Am. J. Clin. Nutr. 93, 601–607. 10.3945/ajcn.110.007369 21177796 PMC3041601

[B54] Valls-Pedret C. Lamuela-Raventós R. M. Medina-Remón A. Quintana M. Corella D. Pintó X.. (2012). Polyphenol-rich foods in the Mediterranean diet are associated with better cognitive function in elderly subjects at high cardiovascular risk. J. Alzheimers. Dis. 29, 773–782. 10.3233/JAD-2012-111799 22349682

[B55] Vazquez-Martin A. Fernández-Arroyo S. Cufí S. Oliveras-Ferraros C. Lozano-Sánchez J. Vellón L.. (2012). Phenolic secoiridoids in extra virgin olive oil impede fibrogenic and oncogenic epithelial-to-mesenchymal transition: extra virgin olive oil as a source of novel antiaging phytochemicals. Rejuvenat. Res. 15, 3–21. 10.1089/rej.2011.1203 22229524 PMC3283896

[B56] Visioli F. Galli C. (1998). Olive oil phenols and their potential effects on human health. J. Agric. Food Chem. 46, 4292–4296. 10.1021/jf980049c

[B57] Visioli F. Bellomo G. Montedoro G. Galli C. (1995). Low density lipoprotein oxidation is inhibited *in vitro* by olive oil constituents. Atherosclerosi 117, 25–32. 10.1016/0021-9150(95)05546-9 8546752

[B58] Visioli F. Caruso D. Galli C. Viappiani S. Galli G. Sala A. (2000a). Olive oils rich in natural catecholic phenols decrease isoprostane excretion in humans. Biochem. Biophys. Res. Commun. 278, 797–799. 10.1006/bbrc.2000.3879 11095986

[B59] Visioli F. Galli C. Bornet F. Mattei A. Patelli R. Galli G.. (2000b). Olive oil phenolics aredose-dependently absorbed in humans. FEBS Lett. 468, 159–160. 10.1016/S0014-5793(00)01216-3 10692578

[B60] Walter W. M. Fleming H. P. Etchells J. L. (1973). Preparation of antimicrobial compounds by hydrolysis of oleuropein from green olives. Appl. Microbiol. 26, 773–776. 4762396 10.1128/am.26.5.773-776.1973 PMC379900

